# Supplementary material for: Single-cell morphological and topological atlas reveals the ecosystem diversity of human breast cancer
Source: Nat Commun. 2023 Oct 25;14:6796. doi: 10.1038/s41467-023-42504-y (PMC10600153; doi:10.1038/s41467-023-42504-y)
Supplement: Supplementary file 1 — Supplementary Information [file 41467_2023_42504_MOESM1_ESM.pdf]

Supplementary Materials

Single-cell morphological and topological atlas reveals the ecosystem diversity of human breast cancer

This PDF file includes:

Supplementary Figure S1 to S14

Supplementary Table S1 to S7

Supplementary Fig. S1

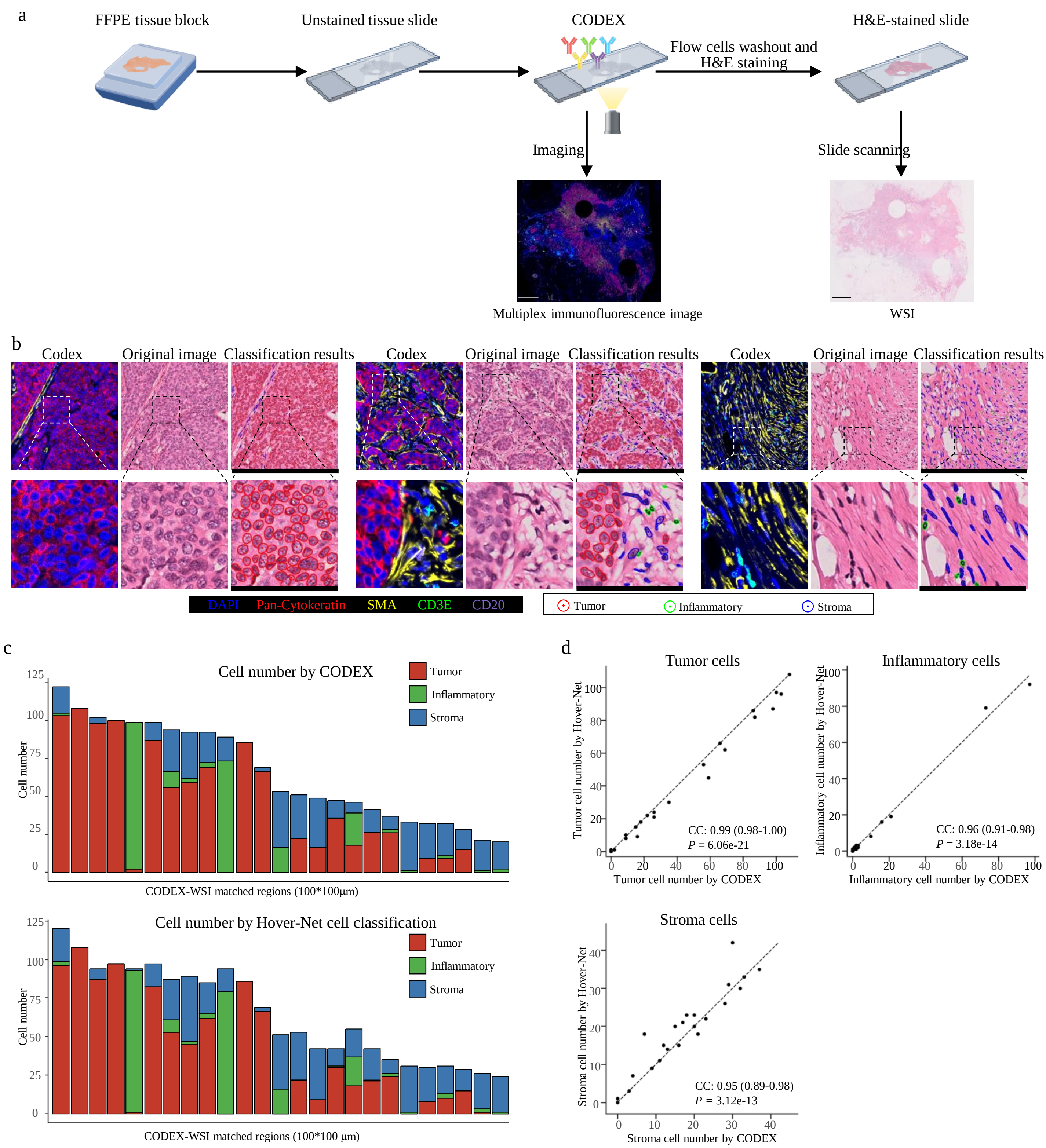

Supplementary Fig. S1. Validation of cell classification accuracy using paired co-detection by indexing (CODEX) and H&E-stained WSIs.

a. Experiment design for paired CODEX and H&E-staining on tissue slides. Created with BioRender.com.

b. Examples of CODEX staining and Hover-Net cell classification results in matched regions. Scale bar for the first row images: 400μm; Scale bar for the second row images: 100μm.

c. The estimated number of tumor, inflammatory and stroma cells through CODEX and Hover-Net cell classification in matched regions (25 randomly selected 100 * 100 μm regions).

d. Comparison of the estimated number of tumor, inflammatory and stroma cells betweeen CODEX and Hover-Net cell classification in matched regions (25 randomly selected 100 * 100 μm regions). Two-sided Spearman correlation coefficient with 95% confidence interval and the corresponding P values are shown.

Abbreviations: FFPE, formalin-fixed paraffin-embedded; CODEX, co-detection by indexing; WSI, whole slide image; CC, correlation coefficient.

Supplementary Fig. S2

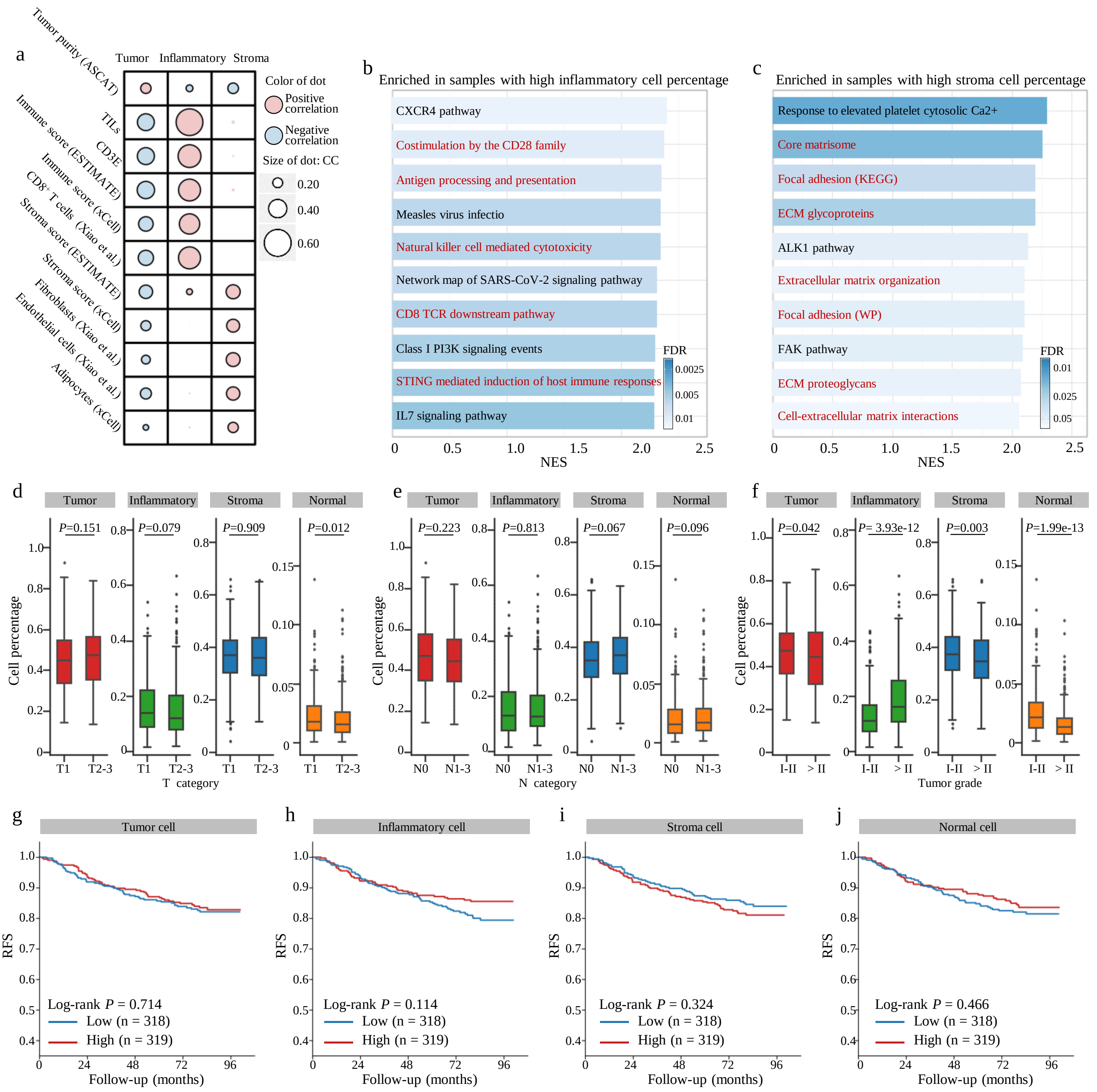

Supplementary Fig. S2. Validation of cell classification results and the correlation between cell composition and patient clinicopathological characteristics and prognosis, related to Fig. 1.

a. Correlation between the tumor, inflammatory and stroma cell percentage with tumor purity estimated by ASCAT, tumor-infiltrating lymphocytes, CD3E mRNA expression, immune score and stroma score from ESTIMATE algorithm, immune score, stroma score and adipocytes from xCell algorithm, and the estimated cell abundance of $CD8^{+}$ T cells, fibroblasts and endothelial cells through single-sample gene set enrichment analysis based on the RNA-seq data. Two-sided Spearman correlation analysis is performed. The size of the dots is proportional to the Spearman correlation coefficient. The outlined dots indicate *P* values < 0.05.

b. Gene sets enriched in the samples with high inflammatory cell percentage. The gene sets associated with immune response are marked in red. The NES and FDR output by GSEA are presented.

c. Gene sets enriched in the samples with high stroma cell percentage. The gene sets associated with extracellular matrix and cell-matrix adhesions are marked in red. The NES and FDR output by GSEA are presented.

d-f. Boxplots of the percentage of tumor, inflammatory, stroma and normal cells of WSIs according to the d) T category, e) N category and f) tumor grade. *P* values are calculated using the two-sided Mann-Whitney U test.
g-j. Kaplan-Meier curves of recurrence-free survival according to the percentage of g) tumor, h) inflammatory,i) stroma and j) normal cells.
Abbreviations: CC, correlation coefficient; NES, normalized enrichment score; FDR, false discovery rate; TILs, tumor-infiltrating lymphocytes; RFS, recurrence-free survival.

Supplementary Fig. S3

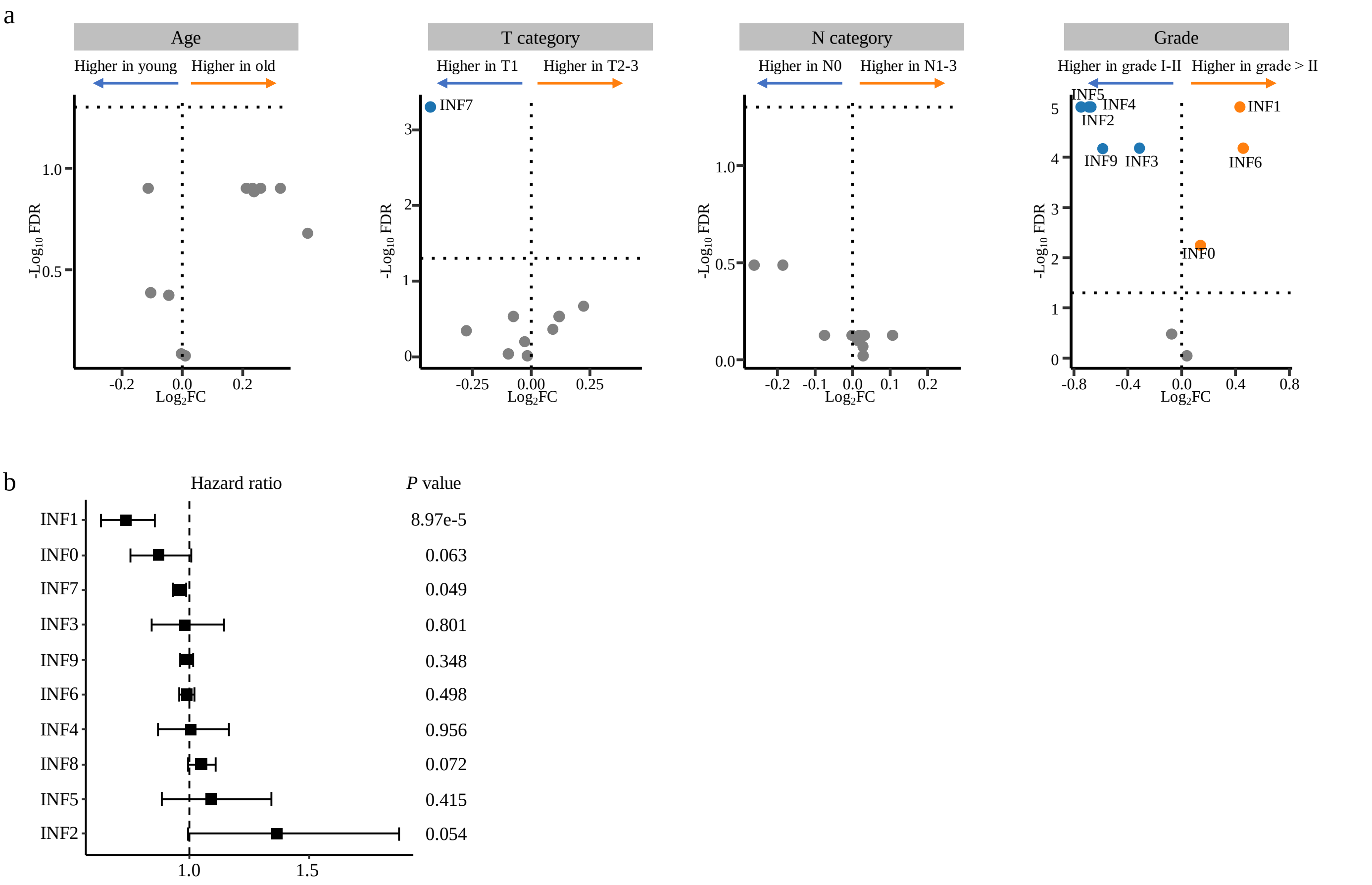

Supplementary Fig. S3. Volcano plot showing the association of inflammatory cell clusters with clinicopathological characteristics and patient prognosis, related to Fig. 2.

a. Volcano plot showing the association of inflammatory cell clusters with clinicopathological characteristics. Fold change is calculated as the ratio of the median percentage value between groups. *P* values are calculated using the two-sided Mann-Whitney U test with false discovery rate-correction for multiple testing. FDR values smaller than 10^{-5} are set to 10^{-5}. The horizontal dotted line indicates an FDR value of 0.05.

b. Forest plot showing the univariate Cox regression analysis of recurrence-free survival for inflammatory cell clusters modeled as log-transformed proportion data (n=637). Squares and whiskers represent point estimates and the 95% confidence interval of hazard ratios.

Abbreviations: FC, fold change; FDR, false discovery rate.

Supplementary Fig. S4

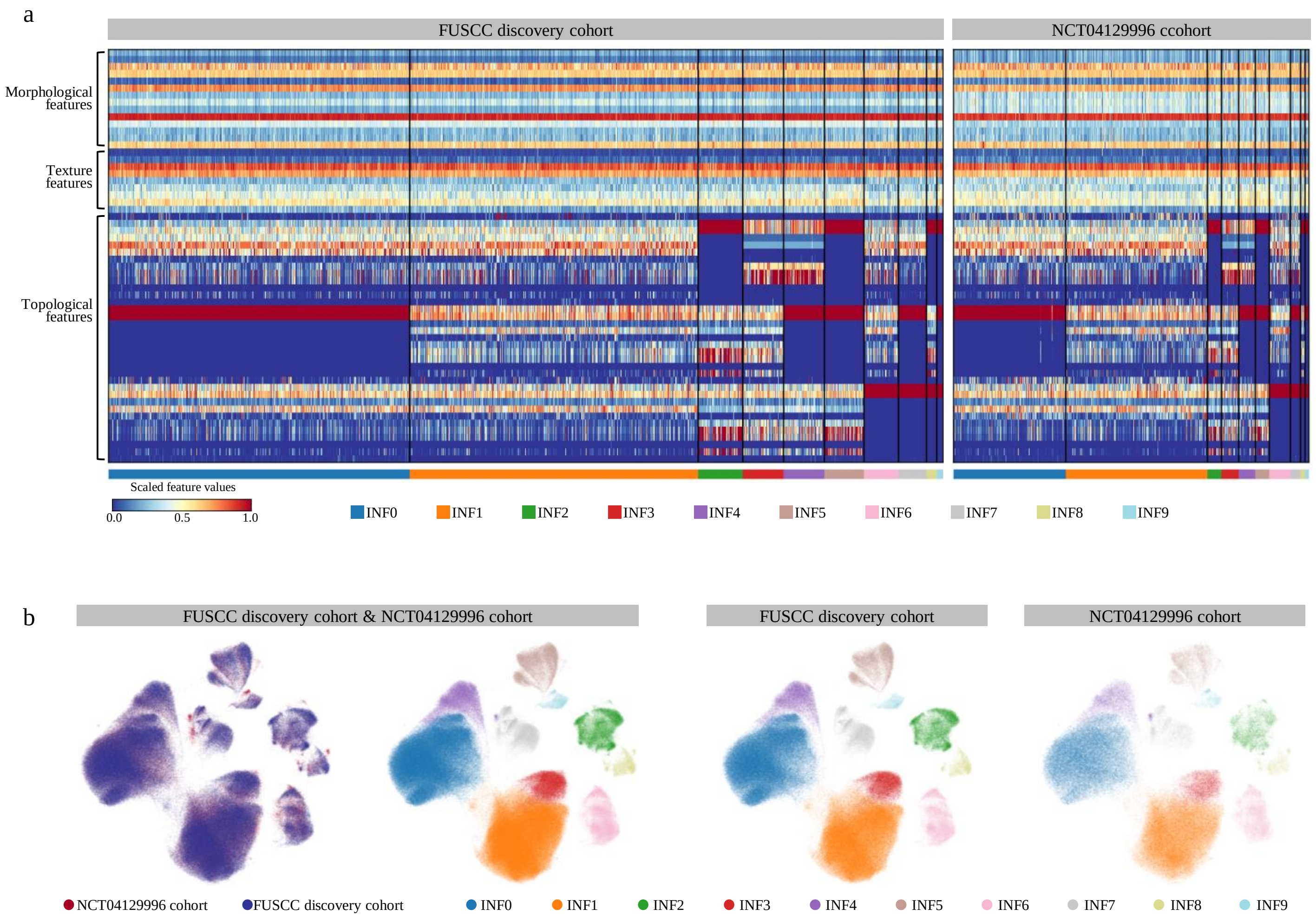

Supplementary Fig. S4. Extrapolation of inflammatory cell clustering from the FUSCC discovery cohort to the NCT04129996 cohort, related to Fig. 2 and Fig. 3.

a. Feature heatmaps show high similarity in the proportions and feature profiles of inflammatory cell clusters between the FUSCC discovery cohort and the NCT04129996 cohort.

b. UMAP plots show high similarity in the embeddings of inflammatory cell clusters between the FUSCC discovery cohort and the NCT04129996 cohort.

Abbreviations: FUSCC, Fudan University Shanghai Cancer Center.

Supplementary Fig. S5

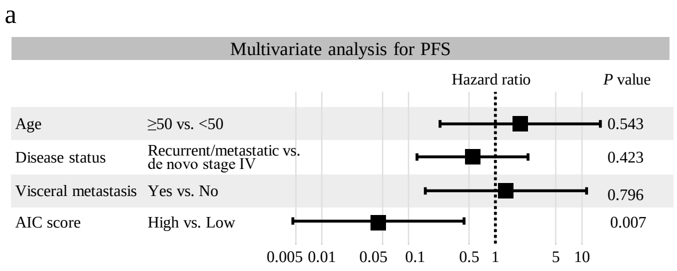

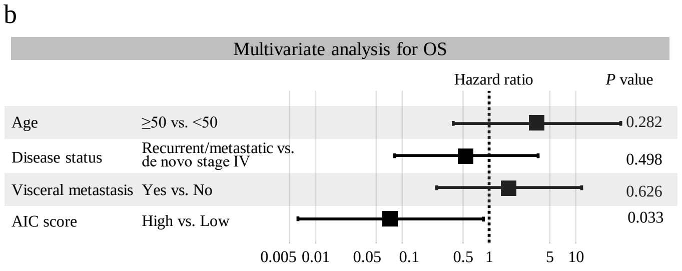

Supplementary Fig. S5. Multivariate Cox analysis of a) PFS and b) OS in the NCT04129996 cohort, related to Fig. 3. Squares and whiskers represent point estimates and the 95% confidence interval of hazard ratios.
Abbreviations: AIC score, aggregated inflammatory cell abundance score; PFS, progression-free survival; OS, overall survival.

Supplementary Fig. S6

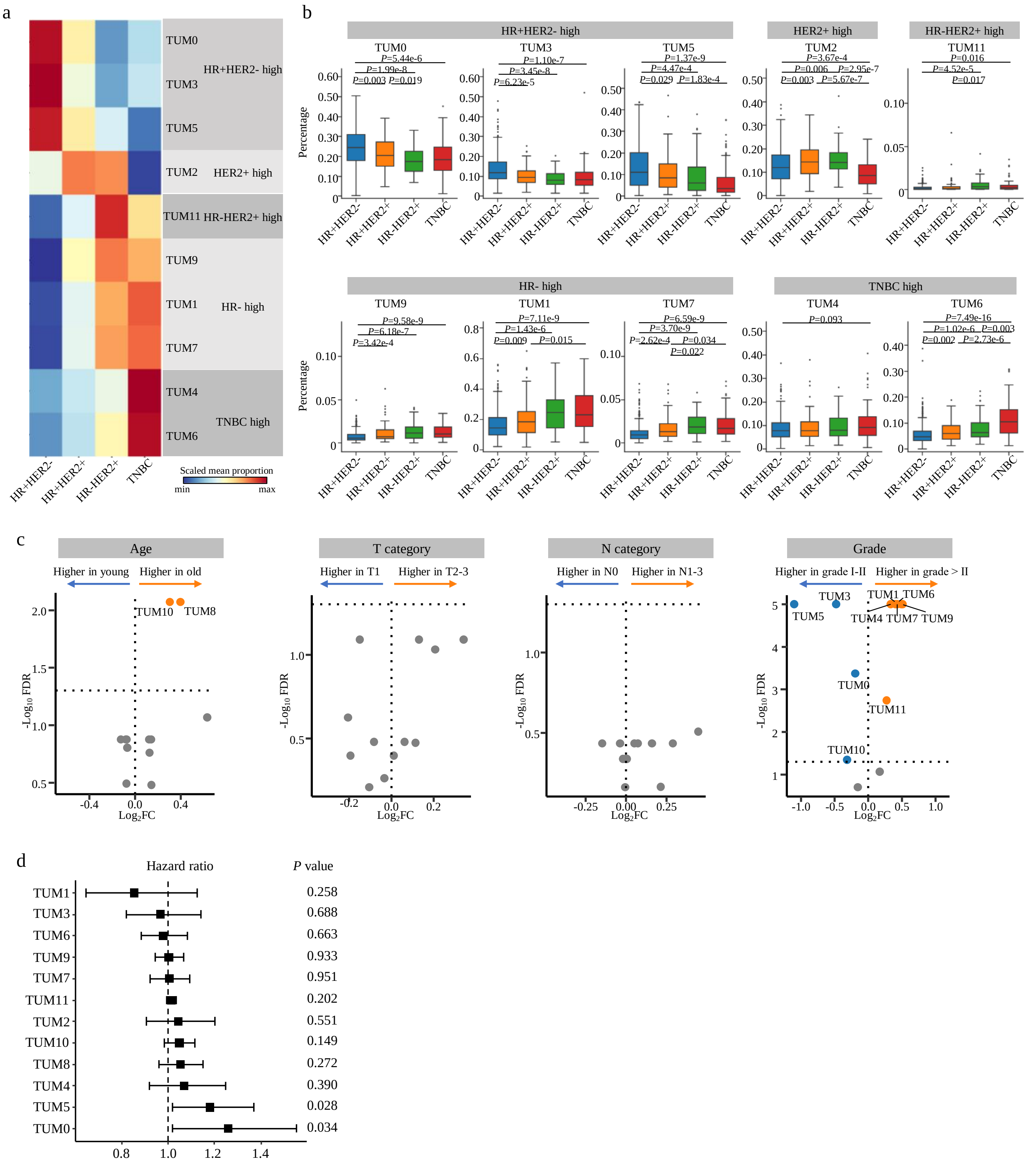

Supplementary Fig. S6. Association of the tumor cell clusters with breast cancer IHC subtypes, clinicopathological characteristics and patient prognosis, related to Fig. 4.

a. Comparison of the abundance of tumor cell clusters among the IHC subtypes. Heatmap shows the scaled mean percentage of tumor cell clusters that are significantly different among the IHC subtypes.

b. The percentage of tumor cell clusters according to the IHC subtypes (HR+HER2-: n=405; HR+HER2+: n=85; HR-HER2+: n=66; TNBC: n=81). *P* values are calculated using the two-sided Mann-Whitney U test with false discovery rate-correction for multiple testing.

c. Volcano plot showing the association of tumor cell clusters with clinicopathological characteristics. Fold change is calculated as the ratio of the median percentage value between groups. *P* values are calculated using the two-sided Mann-Whitney U test with false discovery rate-correction for multiple testing. FDR values smaller than 10^{-5} are set to 10^{-5}. The horizontal dotted line indicates an FDR value of 0.05.
d. Forest plot showing the univariate Cox regression analysis of recurrence-free survival for tumor cell clusters modeled as log-transformed proportion data (n=637). Squares and whiskers represent point estimates and the 95% confidence interval of hazard ratios.
Abbreviations: FC, fold change; FDR, false discovery rate.

Supplementary Fig. S7

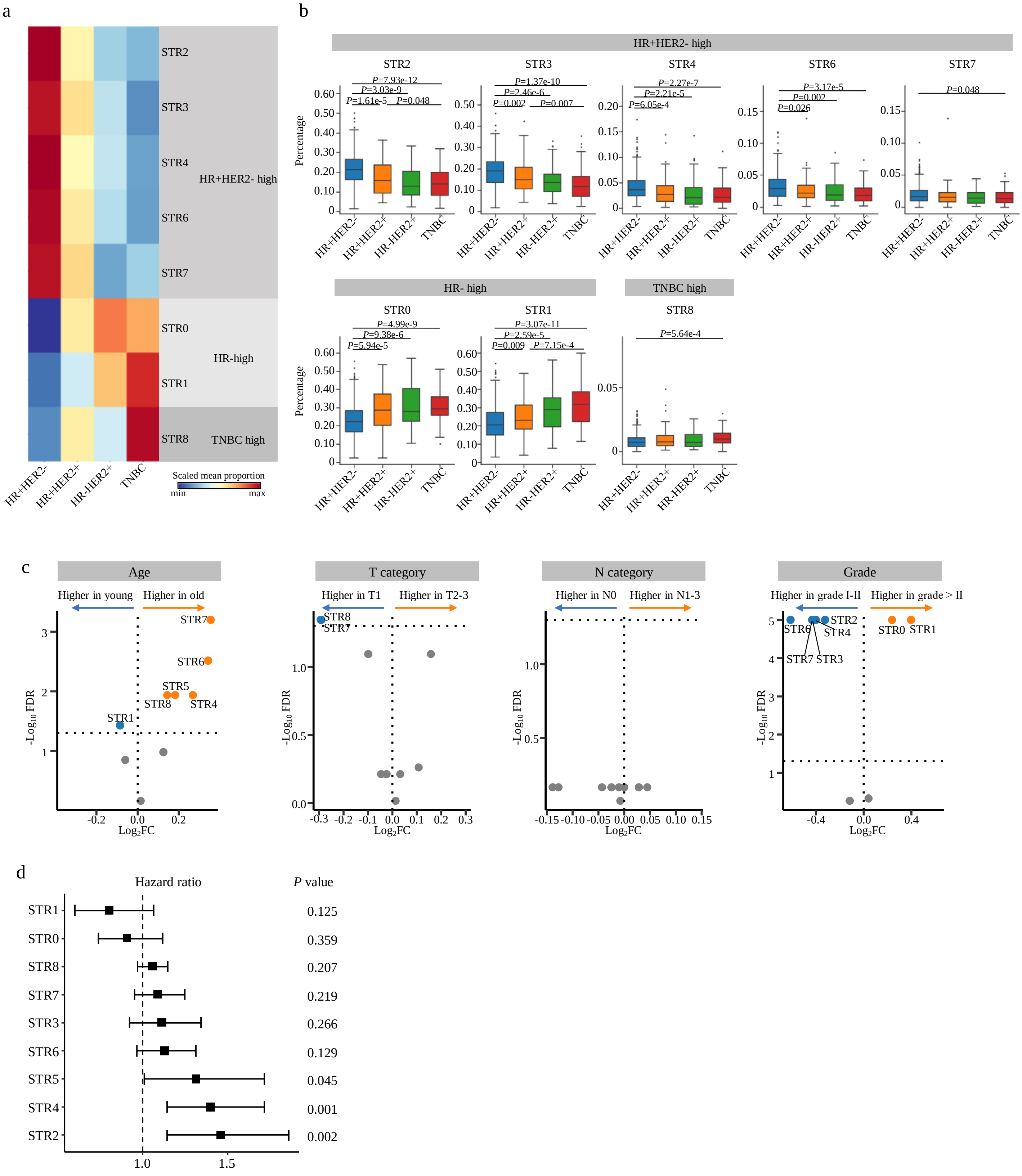

Supplementary Fig. S7. Association of the stroma cell clusters with breast cancer IHC subtypes, clinicopathological characteristics and patient prognosis, related to Fig. 4.

a. Comparison of the abundance of stroma cell clusters among the IHC subtypes. Heatmap shows the scaled mean percentage of stroma cell clusters that are significantly different among the IHC subtypes.

b. The percentage of stroma cell clusters according to the IHC subtypes (HR+HER2-: n=405; HR+HER2+: n=85; HR-HER2+: n=66; TNBC: n=81). *P* values are calculated using the two-sided Mann-Whitney U test with false discovery rate-correction for multiple testing.

c. Volcano plot showing the association of tumor cell clusters with clinicopathological characteristics. Fold change is calculated as the ratio of the median percentage value between groups. *P* values are calculated using the two-sided Mann-Whitney U test with false discovery rate-correction for multiple testing. FDR values smaller than 10^{-5} are set to 10^{-5}. The horizontal dotted line indicates an FDR value of 0.05.
d. Forest plot showing the univariate Cox regression analysis of recurrence-free survival for stroma cell clusters modeled as log-transformed proportion data (n=637). Squares and whiskers represent point estimates and the 95% confidence interval of hazard ratios.
Abbreviations: FC, fold change; FDR, false discovery rate.

Supplementary Fig. S8

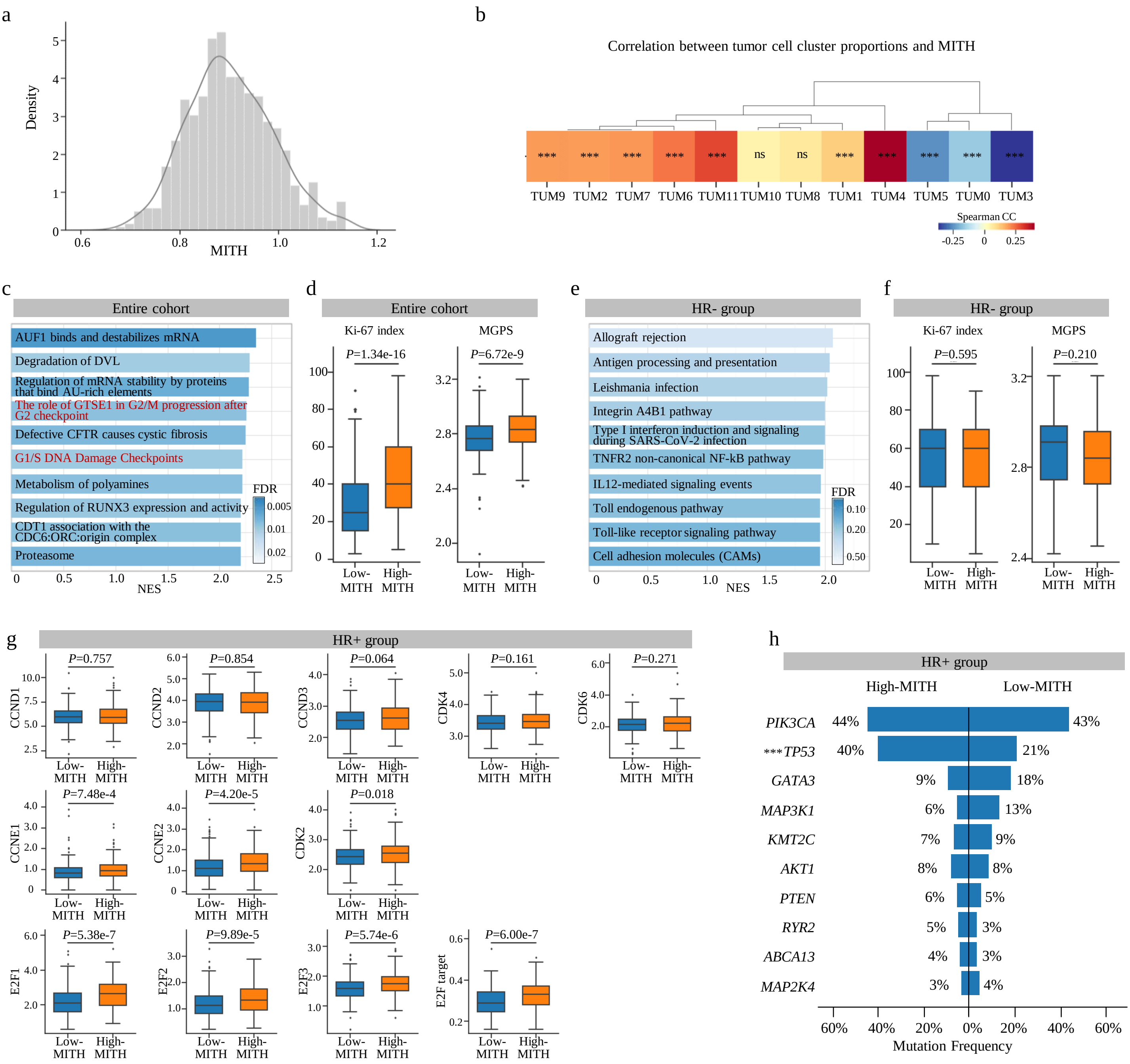

Supplementary Fig. S8. Supplementary data for tumor nuclear morphological intratumor heterogeneity (MITH), related to Fig. 5.

a. Density plot of the MITH values of the FUSCC cohort.

b. Correlation between tumor cell cluster abundance and MITH. Two-sided Spearman correlation coefficient and the false discovery rate-corrected *P* values are shown. ***, *P* < 0.001; ns, not significant.

c. Gene sets enriched in high-MITH samples in the entire cohort revealed by gene sets enrichment analysis. Cell cycle-related gene sets are marked in red. The NES and FDR output by GSEA are presented.

d. Comparison of Ki-67 index and multigene proliferation score between high- and low-MITH samples in the entire cohort. *P* values are calculated using the two-sided Mann-Whitney U test.

e. Gene sets enriched in high-MITH samples in HR- breast cancers revealed by gene sets enrichment analysis. The NES and FDR output by GSEA are presented.

f. Comparison of Ki-67 index and multigene proliferation score between high- and low-MITH samples in HR- breast cancers. *P* values are calculated using the two-sided Mann-Whitney U test.

g. Comparison of mRNA expression of CCND1, CCND2, CCND3, CDK4, CDK6, CCNE1, CCNE2, CDK2, E2F1, E2F2, E2F3 and E2F target signature score between the high- and low-MITH samples in HR+ breast cancers. *P* values are calculated using the two-sided Mann-Whitney U test.

h. Genomic mutation frequencies between high- and low-MITH cases in HR+ breast cancers. The top 10 mutated genes are shown. *P* value is calculated using the chi-square test with false discovery rate-correction for multiple testing. ***, $P < 0.001$.

In Supplementary Fig. S8c-h, high- and low- MITH subgroups are defined based on the median MITH values of the corresponding patient groups.

Abbreviations: MITH, morphological intratumor heterogeneity; CC, correlation coefficient; NES, normalized enrichment score; FDR, false discovery rate; MGPS, multigene proliferation score.

Supplementary Fig. S9

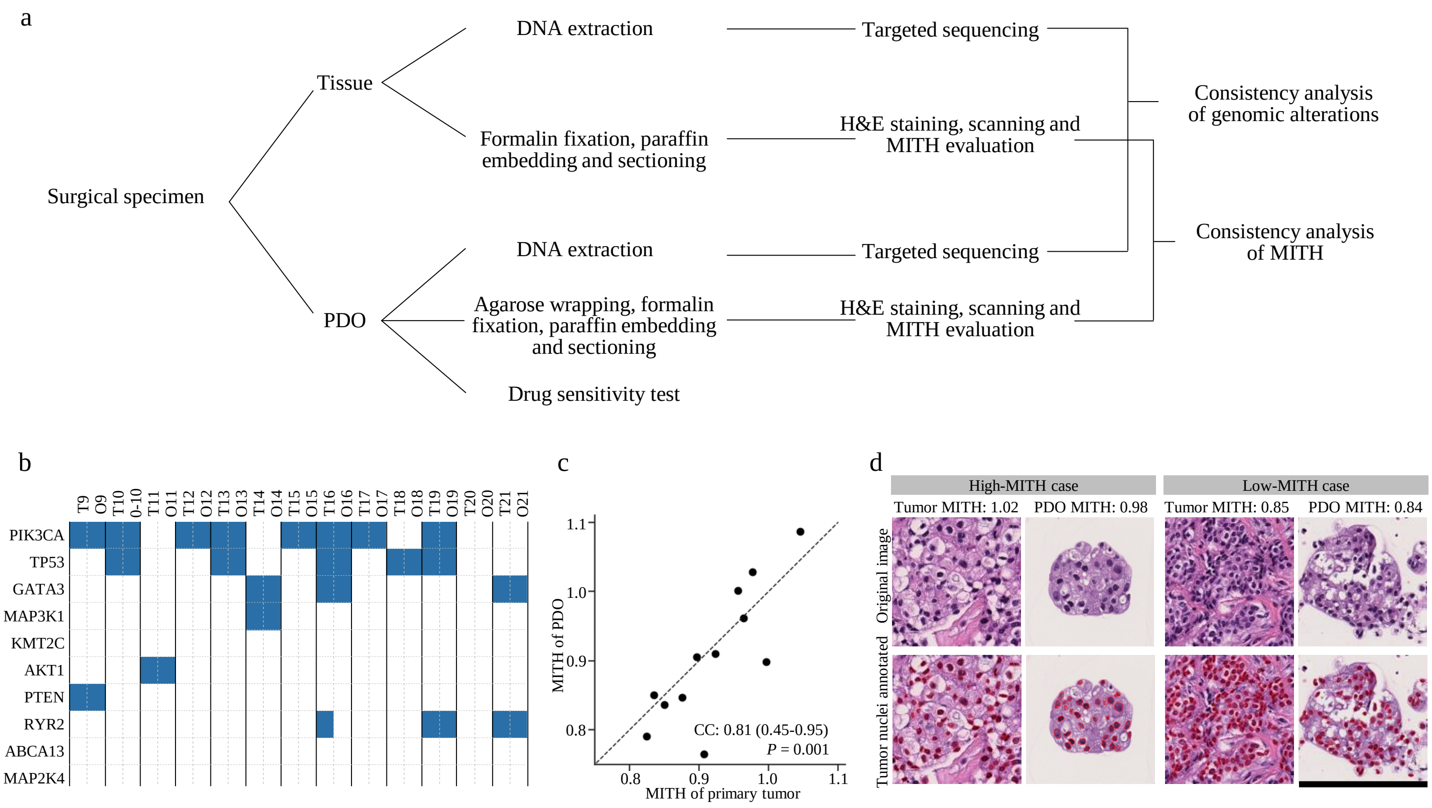

Supplementary Fig. S9. Comparison between the primary tumors and the corresponding PDOs in genomic alterations and MITH, related to Fig. 5.

a. Diagram of examining the consistency between the primary tumors and the corresponding PDOs in genomic alterations and MITH.

b. Comparison of genomic alterations between the primary tumors and the corresponding PDOs (T, primary tumors; O, PDOs).

c. Comparison of MITH values between the primary tumors and the corresponding PDOs. Two-sided Pearson correlation coefficient with 95% confidence interval and the corresponding *P* value are shown.

d. Local images of the primary tumors and the corresponding PDOs. Scale bar: 200μm.

Abbreviations: MITH, morphological intratumor heterogeneity; PDO, patient-derived organoid; CC, correlation coefficient.

Supplementary Fig. S10

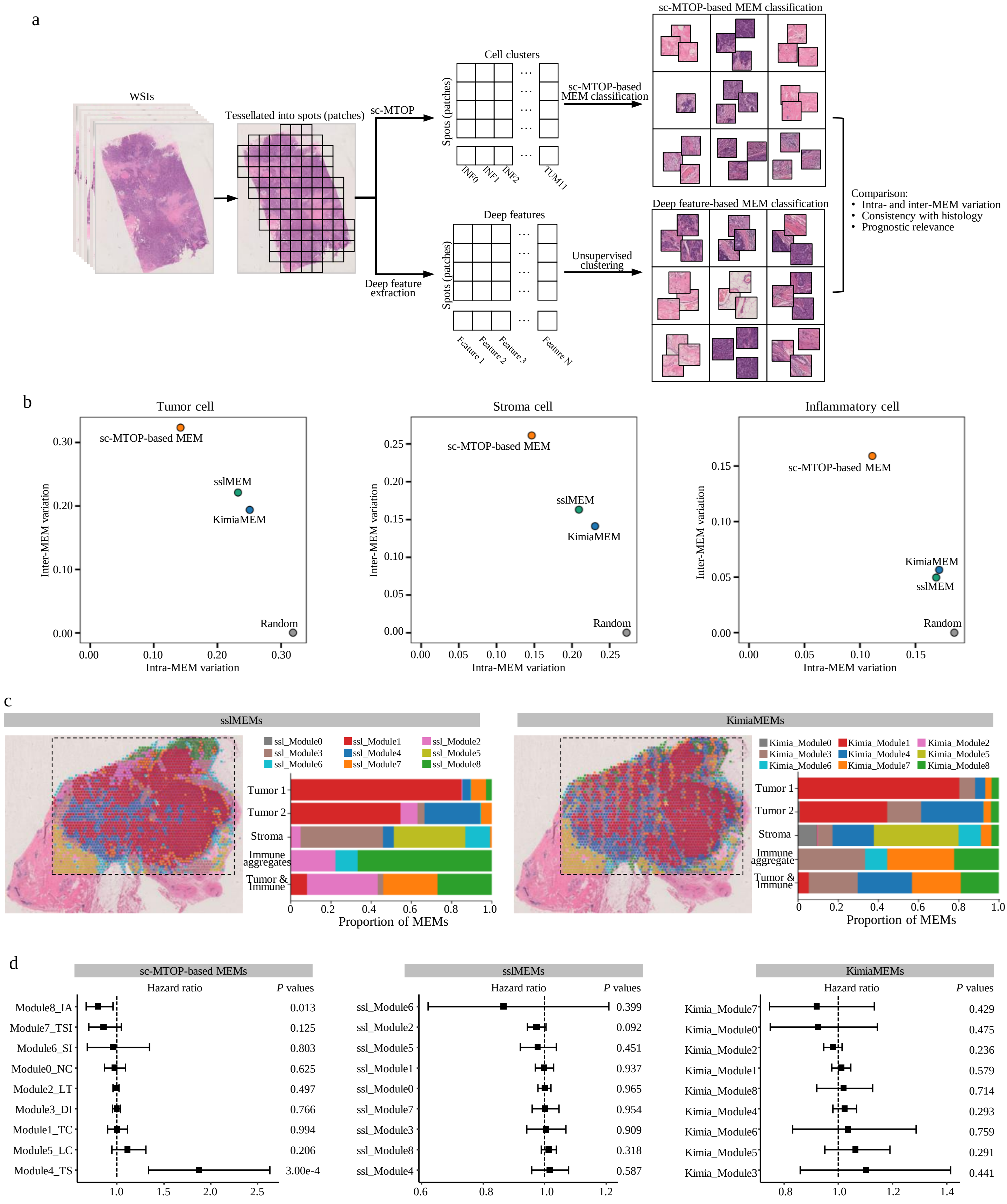

Supplementary Fig. S10. Comparison between the deep learning-based method and our sc-MTOP-based method in characterizing the breast cancer ecosystem and providing clinically relevant information., related to Fig. 6.

a. Diagram for the comparion between the deep learning-based method and our sc-MTOP-based method.

b. The intra- and inter-MEM variation of the tumor, stroma and inflammatory cell proportions for the sc-MTOP-based MEMs, sslMEMs and KimiaMEMs. A random nine-class classification was used as a negative control for this analysis.

c. Thumbnail of a whole slide image with sslMEM mapping and KimiaMEM mapping. The MEM compositions of different histological regions are presented aside (also see Fig.6e).

d. Forest plot showing the univariate Cox regression analysis of recurrence-free survival for sc-MTOP-based MEMs, sslMEMs and KimiaMEMs modeled as log-transformed module percentages (n=637). Squares and whiskers represent point estimates and the 95% confidence interval of hazard ratios.

Abbreviations: WSI, whole slide image; sc-MTOP, single-cell morphological and topological profiling; MEM, micro-ecological module; sslMEM, self-supervised learning model-based micro-ecological module; KimiaMEM, KimiaNet-based micro-ecological module; RFS, recurrence-free survival.

Supplementary Fig. S11

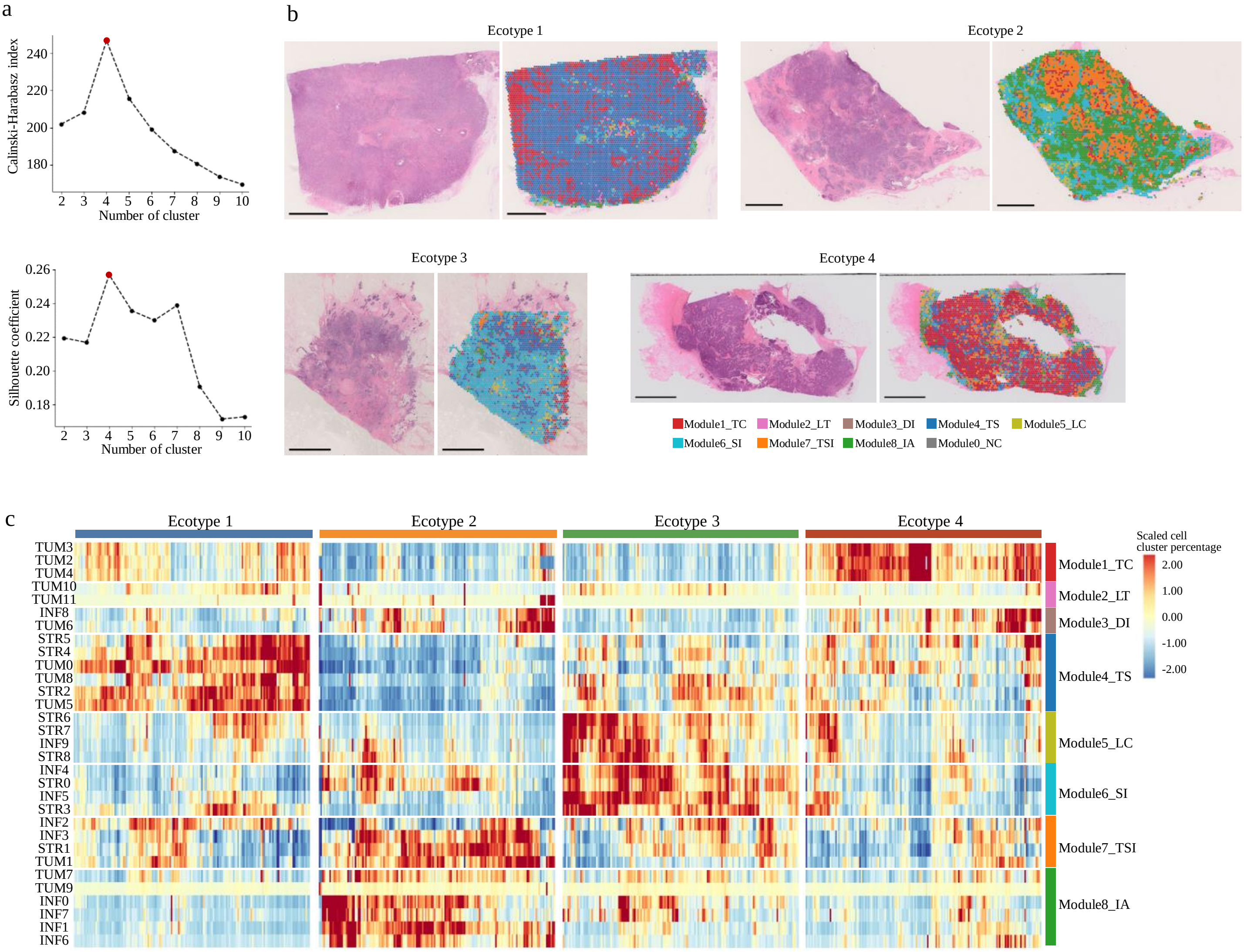

Supplementary Fig. S11. Supplementary data for micro-ecological module-based breast cancer ecotypes, related to Fig. 7.
a. Selection of cluster number for hierarchical clustering based on Calinski-Harabasz index and Silhouette coefficient.
b. An example of each ecotype. The thumbnail of raw whole slide images and the embedding of micro-ecological modules are shown. Scale bar: 4mm.
c. Cell cluster profiles according to the breast cancer ecotypes. Rows are ordered according to the MEMs.
Abbreviations: MEM, micro-ecological module.

Supplementary Fig. S12

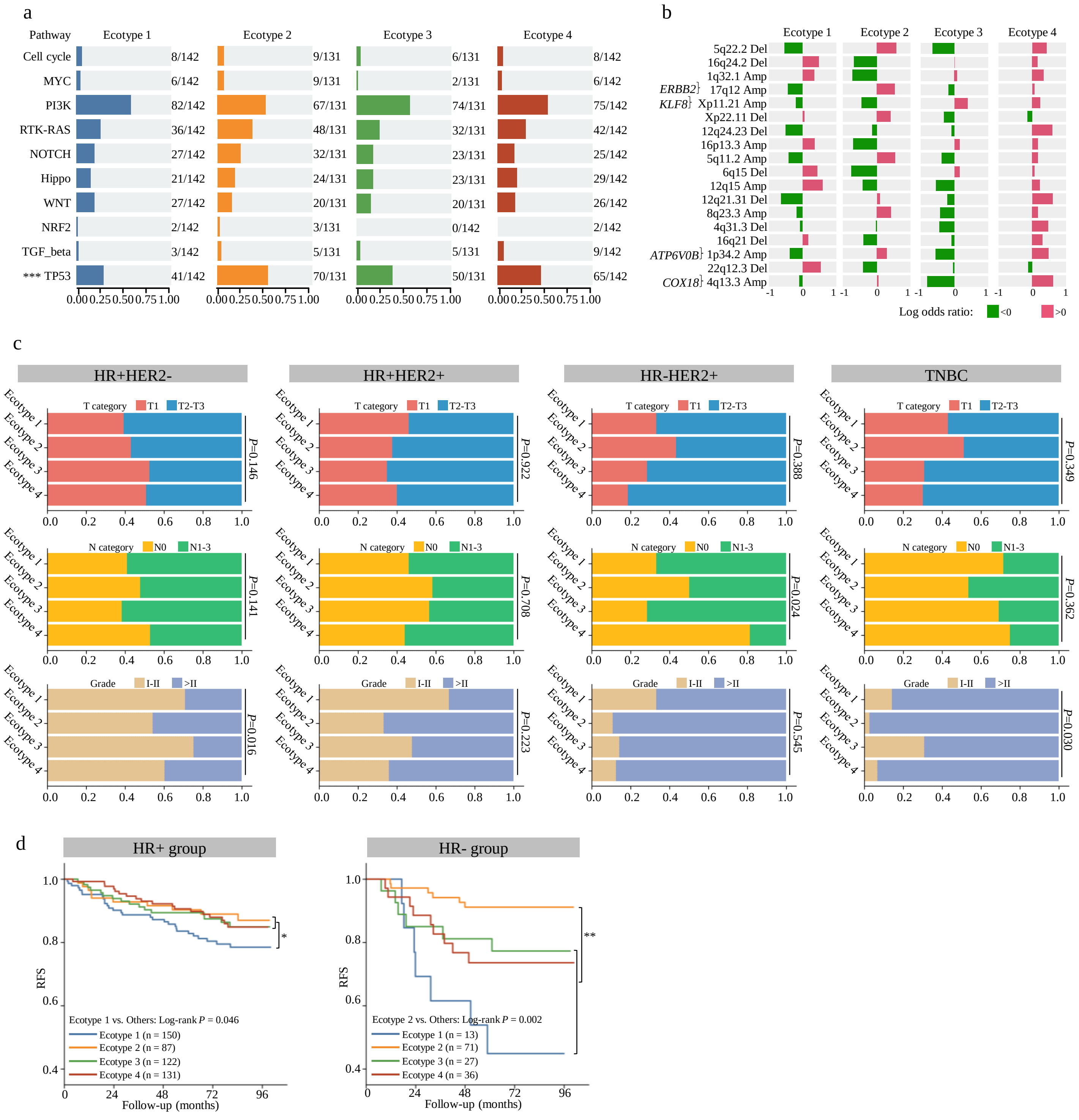

Supplementary Fig. S12. Supplementary data for the correlation of breast cancer ecotypes with genomic alterations and clinicopathological characteristics, related to Fig. 7.

a. Enrichment of oncogenic pathway alterations among ecotypes. The fractions of patients with altered pathway are shown and compared across the four ecotypes. *P* values are calculated using the chi-square test. ***, $P < 0.001$.

b. Enrichment of copy number alterations (CNAs) among ecotypes. Log10 odds ratio was used to measure the effect of ecotypes on CNA events. CNA events that show significant difference in enrichment among the four ecotypes are shown. Del, deletion; Amp, amplification.

c. Association between breast cancer ecotypes and tumor T category, N category and grade within each IHC subtype. *P* values are calculated using the chi-square test.

d. Kaplan-Meier curves of RFS for ecotypes in HR+ and HR- breast cancer patients. **, $P < 0.01$; *, $P < 0.05$.

Abbreviations: RFS, recurrence-free survival.

Supplementary Fig. S13

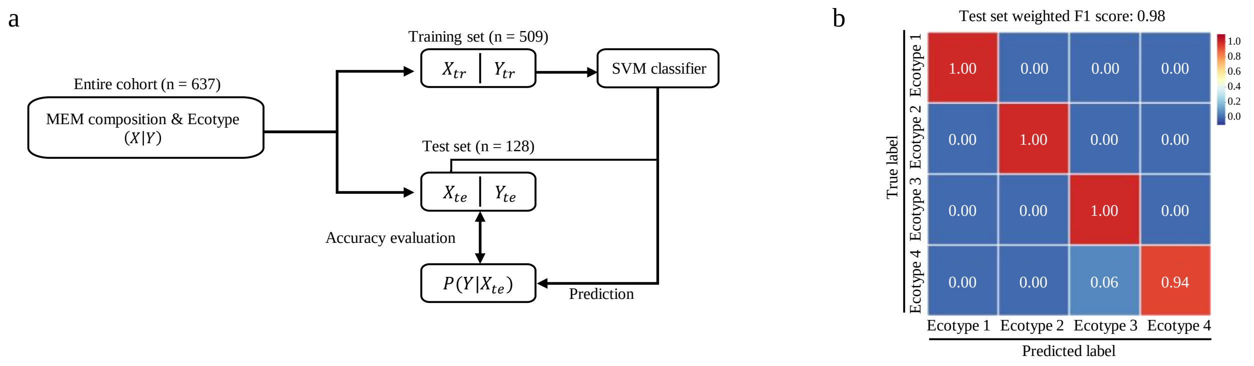

Supplementary Fig. S13. Development of a breast cancer ecotype classifier

a. Diagram for developing a breast cancer ecotype classifier to distinguish samples' tumor ecotypes using their MEM composition as input.

b. Confusion matrix of ecotype classification results in the test set.

Abbreviations: SVM, support vector machine.

Supplementary Fig. S14

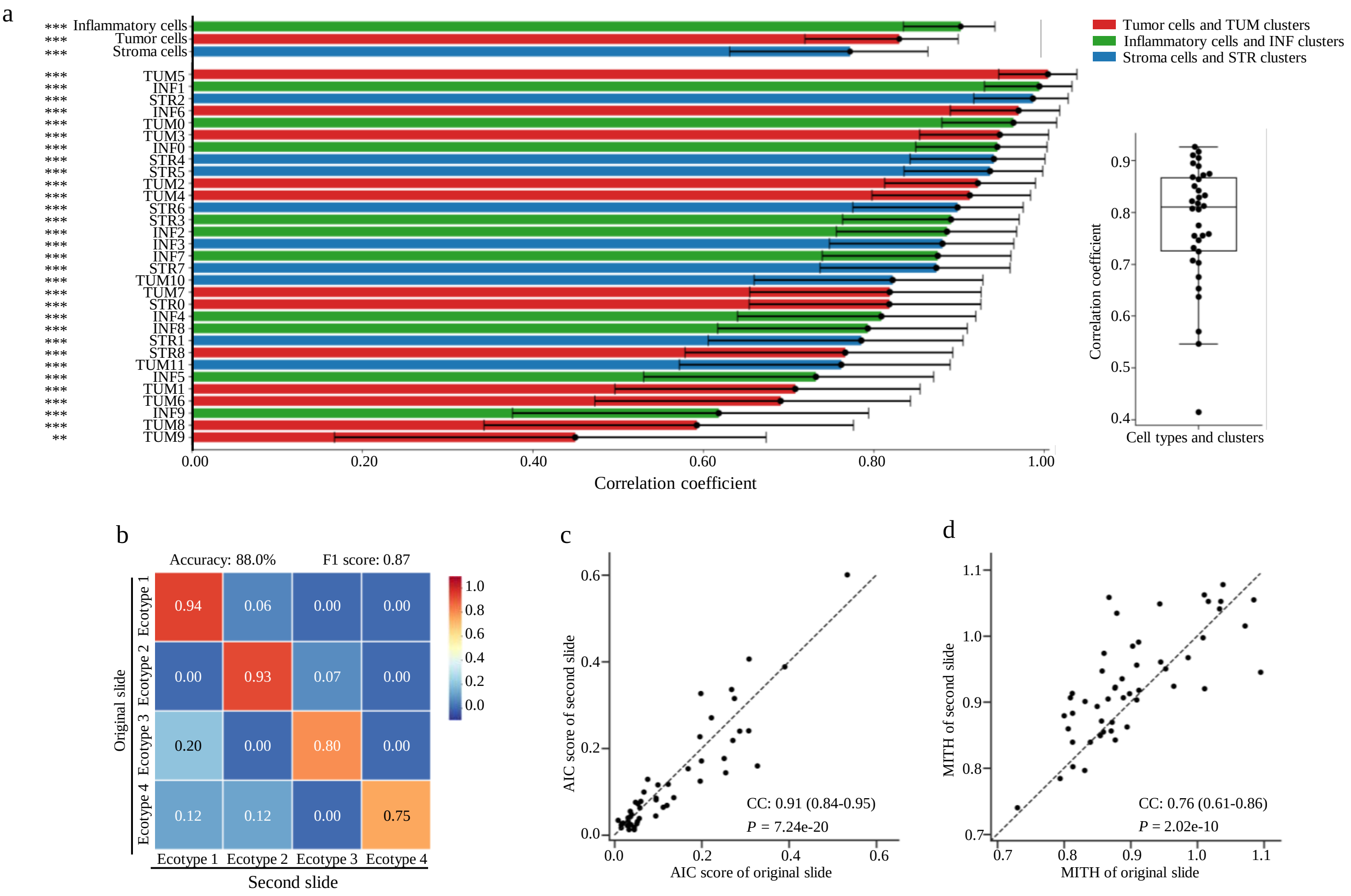

Supplementary Fig. S14. Reproducibility evaluation of our sc-MTOP algorithm on a second slide of the same patient.

a. Barplot and boxplot (inset) showing the correlation of the percentage of major cell types and cell clusters (TUM, STR and INF) between the original slide and the second slide from the same patient. Two-sided Spearman correlation analysis is performed with *P* values corrected for multiple testing. ***, $P < 0.001$; **, $P < 0.01$. Inset box plot displays the distribution of correlation coefficients. The center line of boxplot indicates the median values; box limits show upper and lower quartiles; whiskers extend from box limits to the farthest data point within 1.5 × interquartile range; points beyond whiskers are outliers.

b. Confusion matrix of the ecotype classification results for the original slide and the second slide of the same patient.

c. Comparison of the AIC score between the original slide and the second slide from the same patient (n=50). Two-sided Spearman correlation coefficient with 95% confidence interval and the corresponding *P* values are shown.

d. Comparison of MITH between the original slide and the second slide from the same patient (n=50). Two-sided Spearman correlation coefficient with 95% confidence interval and the corresponding *P* values are shown.

Abbreviations: AIC score, aggregated inflammatory cell abundance score; MITH, morphological intratumor heterogeneity; CC, correlation coefficient.

Supplementary Tables

Supplementary Table S1. Clinicopathological characteristics of the patients in the FUSCC discovery cohort.

	FUSCC discovery cohort (n = 637)
Age, year	
< 50	264 (41.4)
≥ 50	373 (58.6)
T category	
T1	276 (43.3)
T2	353 (55.4)
T3	8 (1.3)
T4	0 (0)
N category	
N0	309 (48.5)
N1	189 (29.7)
N2	80 (12.6)
N3	59 (9.3)
Tumor grade	
I-II	311 (48.8)
> II	298 (46.8)
Unknown	28 (4.4)
IHC subtype	
HR+HER2-	405 (63.6)
HR+HER2+	85 (13.3)
HR-HER2+	66 (10.4)
TNBC	81 (12.7)
Surgery type	
non-BCS	633 (99.4)
BCS	4 (0.6)
Radiotherapy	
No	400 (62.8)
Yes	177 (27.8)
Unknown	60 (9.4)
Chemotherapy	
No	86 (13.5)
Yes	512 (80.4)
Unknown	39 (6.1)

Abbreviations: IHC, immunohistochemistry; TNBC, triple-negative breast cancer; BCS, breast conserving surgery.
Note: Data are presented as number (percentage) of patients.

Supplementary Table S2. Clinical characteristics of the patients in the NCT04129996 cohort.

Patient ID	Age	Disease status	Visceral metastasis	TILs	CD8 IHC score	PD-L1 IHC score	Response	OS event	OS (Months)
C002	29	de novo stage IV	Yes	20	20%	3%	Non-responder	1	17.2
C007	51	Recurrent/metastatic	Yes	10	20%	5%	Responder	1	8.5
C009	50	de novo stage IV	Yes	70	30%	NA	Responder	0	20.5
C014	63	de novo stage IV	Yes	60	30%	30%	Responder	0	18.7
C015	60	de novo stage IV	Yes	50	25%	NA	Responder	0	18.6
C016	57	Recurrent/metastatic	No	2	10%	NA	Responder	0	18.6
C020	53	Recurrent/metastatic	Yes	10	10%	0%	Responder	1	14.3
C021	31	de novo stage IV	No	10	30%	0%	Responder	0	17.8
C022	38	de novo stage IV	Yes	10	20%	NA	Responder	1	17.0
C026	58	Recurrent/metastatic	No	30	20%	5%	Responder	0	17.1
C031	38	Recurrent/metastatic	Yes	5	20%	5%	Non-responder	0	16.3
C032	59	Recurrent/metastatic	Yes	40	30%	1%	Responder	0	16.3
C033	70	Recurrent/metastatic	No	70	15%	20%	Responder	0	16.1
C034	46	Recurrent/metastatic	Yes	10	20%	20%	Responder	0	16.0
C035	63	de novo stage IV	No	20	20%	5%	Non-responder	1	5.5
C036	40	Recurrent/metastatic	Yes	40	20%	1%	Non-responder	1	4.2
C038	64	de novo stage IV	Yes	20	10%	40%	Responder	0	14.6
C041	27	de novo stage IV	Yes	30	20%	40%	Responder	0	13.2
C042	42	Recurrent/metastatic	Yes	15	10%	60%	Responder	0	13.2
C043	38	de novo stage IV	Yes	30	10%	10%	Responder	0	13.0
C044	42	Recurrent/metastatic	No	50	30%	50%	Responder	0	12.9
C045	60	de novo stage IV	No	20	30%	5%	Responder	1	6.1
C046	49	de novo stage IV	Yes	10	10%	NA	Responder	0	12.6
C047	52	de novo stage IV	Yes	20	30%	NA	Responder	0	12.6

Abbreviations: TILs, tumor-infiltrating lymphocytes; IHC, immunohistochemistry; OS, overall survival; NA, not available.

Supplementary Table S3. Multivariate analysis of recurrence-free survival using Cox proportional hazards models (n=537).

	Recurrence-free survival	
Variables	HR (95% CI)	*P* value
Age		
<50	Reference	-
⩾50	1.55(0.99 - 2.44)	0.056
T category		
T1	Reference	-
T2-3	1.11 (0.70-1.75)	0.660
N category		
N0	Reference	-
N1-3	2.06 (1.17 - 3.62)	0.012
Grade		
grade I-II	Reference	-
grade > II	1.25 (0.77 – 2.02)	0.371
IHC subtype		
TNBC	Reference	-
HR+HER2-	0.41 (0.21 - 0.79)	0.008
HR+HER2+	0.27 (0.10 - 0.71)	0.008
HR-HER2+	0.57 (0.25 - 1.30)	0.181
Ecotypes		
Ecotype 1	Reference	-
Ecotype 2	0.32 (0.16 - 0.62)	8.60e-4
Ecotype 3	0.50 (0.28 - 0.89)	0.018
Ecotype 4	0.59 (0.34- 1.04)	0.070
Chemotherapy		
No	Reference	-
Yes	0.89 (0.44 – 1.79)	0.748
Radiotherapy		
No	Reference	-
Yes	1.63 (0.96 – 2.77)	0.069

Abbreviations: TNBC, triple-negative breast cancer; IHC, immunohistochemistry; HR, hazard ratio; CI, confidence interval.

Supplementary Table S4. Morphological, texture and topological features extracted by single-cell morphological and topological profiling.

Feature name	Category	Description	Algorithm
Area	Morphological	Number of pixels of the nuclear region.	skimage.measure.regionprops.area
AreaBbox	Morphological	Number of pixels of the nuclear bounding box.	skimage.measure.regionprops.bbox_area
CellEccentricities	Morphological	Eccentricity of the ellipse that has the same second-moments as the nuclear region. The eccentricity is the ratio of the focal distance (distance between focal points) over the major axis length.	skimage.measure.regionprops.eccentricity
Circularity	Morphological	The measurement of the roundness of the nuclear region. The circularity is defined by the ratio of the 4 pi times the area over the square of the perimeter.	(4*np.pi*skimage.measure.regionprops.area)/(skimage.measure.regionprops.perimeter**2)
Elongation	Morphological	Ratio of the major axis length over the minor axis length.	skimage.measure.regionprops.major_axis_length/skimage.measure.regionprops.minor_axis_length
Extent	Morphological	Ratio of pixels in the region to pixels in the total bounding box.	skimage.measure.regionprops.extent
MajorAxisLength	Morphological	The length of the major axis of the ellipse that has the same normalized second central moments as the nuclear region.	skimage.measure.regionprops.major_axis_length
MinorAxisLength	Morphological	The length of the minor axis of the ellipse that has the same normalized second central moments as the nuclear region.	skimage.measure.regionprops.minor_axis_length

Perimeter	Morphological	Perimeter of object which approximates the nuclear contour to the equivalent polygon using 4-connectivity lines.	skimage.measure.regionprops.perimeter
Solidity	Morphological	Ratio of pixels in the region to pixels of the convex hull image.	skimage.measure.regionprops.solidity
CurvMean	Morphological	The mean of all anchor point's curvature. The anchor point is taken from the contour of the nuclear at intervals.	Implemented according to https://github.com/popellab/MIBC-Predictive-models
CurvMax	Morphological	The maximum of all anchor point's curvature. The anchor point is taken from the contour of the nuclear at intervals.	Implemented according to https://github.com/popellab/MIBC-Predictive-models
CurvMin	Morphological	The minimum of all anchor point's curvature. The anchor point is taken from the contour of the nuclear at intervals.	Implemented according to https://github.com/popellab/MIBC-Predictive-models
CurvMstd	Morphological	The standard deviation of all anchor point's curvature. The anchor point is taken from the contour of the nuclear at intervals.	Implemented according to https://github.com/popellab/MIBC-Predictive-models
ASM	Texture	The angular second moment of the co-occurrence matrix.	skimage.feature.graycoprops(GLCM, ‘ASM’)
Contrast	Texture	The sum of squares variances of the co-occurrence matrix.	skimage.feature.graycoprops(GLCM, ‘contrast’)
Correlation	Texture	The correlation texture measures the linear dependency of grey levels on those of neighboring pixels.	skimage.feature.graycoprops(GLCM, ‘correlation’)

Entropy	Texture	The opposite of ASM, measures irremediable chaos or disorder.	Implemented according to https://murphylab.web.cmu.edu/publications/boland/boland_node26.html
Homogeneity	Texture	The inverse difference moment of the co-occurrence matrix.	skimage.feature.graycoprops(GLCM, 'homogeneity)
IntensityMean	Texture	The mean of intensity value of nuclear image.	Calculated according to the description
IntensityStd	Texture	The standard deviation of intensity value of nuclear image.	Calculated according to the description
IntensityMax	Texture	The maximum of intensity value of nuclear image.	Calculated according to the description
IntensityMin	Texture	The minimum of intensity value of nuclear image.	Calculated according to the description
Nsubgraph	Topological	The number of nuclei in the subgraph where the nucleus is located.	igraph.Graph().vcount()
Degrees	Topological	The number of edges connected with the nucleus.	igraph.Graph().degree()
Coreness	Topological	The coreness of a nucleus is k if it is a member of the k-core but not a member of the (k+1)-core.	igraph.Graph().coreness()

Eccentricity	Topological	The maximum of the shortest distance from this nucleus to all other nuclei in the graph.	igraph.Graph().eccentricity()
Eccentricity_normed	Topological	The normalized value of the eccentricity with the number of the nuclei in the subgraph.	igraph.Graph().eccentricity()/n
Harmonic Centrality	Topological	The harmonic centrality of a nucleus measures how easily other nuclei can be reached from it. It is computed by the mean inverse distance to all other nuclei.	igraph.Graph().harmonic_centrality()
Closeness	Topological	The closeness centrality of a nucleus measures how easily other nuclei can be reached from it. It is computed by the number of nuclei minus one divided by the sum of the lengths of all geodesics from/to the given nucleus.	igraph.Graph().closeness()
Betweenness	Topological	The betweenness is how important role the nucleus plays in the graph. It is computed by the ratio of the number of the shortest paths that pass the nucleus over the number of paths that pass the nucleus in the subgraph.	igraph.Graph().betweenness()
Betweenness _normed	Topological	The normalized value of the betweenness with the number of the nuclei in the subgraph.	igraph.Graph().betweenness()/((n-1)*(n-2) /2)
Clustering Coefficient	Topological	The clustering coefficient is the probability that two neighbors of a nucleus are connected and form a loop.	igraph.Graph().transitivity_local_undirect ed()
MinEdgeLength	Topological	The minimum length of the edges of the nucleus. If there is no edge connected with the nucleus, the value is set as 100 pixels, which is the upper limit of distance between two connected nuclei.	Calculated according to the description
MeanEdgeLength	Topological	The mean length of the edges of the nucleus. If there is no edge connected with the nucleus, the value is set as 100 pixels, which is the upper limit of distance between two connected nuclei.	Calculated according to the description

StromaBlocker	Topological	For each inflammatory nucleus and its closest tumor nucleus, calculate the number of stroma nucleus whose distance to these two nuclei are both smaller than the distance between this inflammatory nucleus and its closest tumor nucleus. The feature is only for inflammatory nucleus.	Calculated according to the description

Supplementary Table S5. CODEX antibody panel.

Product	Manufacturer	Catalog #
Anti-Hu CD3e (AKYP0062)-BX045—Alexa Fluor™ 647 for PhenoCycler	Akoya Biosciences	4550119
Anti-Hu CD4 (AKYP0048)-BX003—Alexa Fluor™ 647 for PhenoCycler	Akoya Biosciences	4550112
Anti-Hu CD8 (AKYP0028)-BX026—Atto 550 for PhenoCycler	Akoya Biosciences	4250012
Anti-Hu CD11c (AKYP0051)-BX024—Alexa Fluor™ 647 for PhenoCycler	Akoya Biosciences	4550114
Anti-Hu CD20 (AKYP0049)-BX007—Alexa Fluor™ 750 for PhenoCycler	Akoya Biosciences	4450018
Anti-Hu CD31 (AKYP0047)-BX001—Alexa Fluor™ 750 for PhenoCycler	Akoya Biosciences	4450017
Anti-Hu CD45RO (AKYP0059)-BX017—Atto 550 for PhenoCycler	Akoya Biosciences	4250023
Anti-Hu CD68 (AKYP0050)-BX015—Alexa Fluor™ 647 for PhenoCycle	Akoya Biosciences	4550113
Anti-Hu CD163 (AKYP0114)-BX069—Atto 550 for PhenoCycler	Akoya Biosciences	4250079
Anti-Hu FOXP3 (AKYP0102)-BX031—Alexa Fluor™ 647 for PhenoCycler	Akoya Biosciences	4550071
Anti-Hu Granzyme B (AKYP0086)-BX041—Atto 550 for PhenoCycler	Akoya Biosciences	4250055
Anti-Hu HLA-DR (AKYP0063)-BX033—Alexa Fluor™ 647 for PhenoCycler	Akoya Biosciences	4550118
Anti-Hu/Mu Ki67 (AKYP0052)-BX047—Atto 550 for PhenoCycler	Akoya Biosciences	4250019
Anti-Hu LAG3 (AKYP0089)-BX055—Alexa Fluor™ 647 for PhenoCycler	Akoya Biosciences	4550058
Anti-Hu Pan-Cytokeratin (AKYP0053)-BX019—Alexa Fluor™ 750 for PhenoCycler	Akoya Biosciences	4450020
Anti-Hu PD-1 (AKYP0070)-BX046—Alexa Fluor™ 647 for PhenoCycler	Akoya Biosciences	4550038
Anti-Hu PD-L1 (AKYP0103)-BX043—Alexa Fluor™ 647 for PhenoCycler	Akoya Biosciences	4550072
Anti-Hu SMA (AKYP0081)-BX013—Alexa Fluor™ 750 for PhenoCycler	Akoya Biosciences	4450049

Supplementary Table S6. Tumor microenvironment cell subsets estimated based on RNA-seq data through ssGSEA.

Tumor microenvironment cell subsets	Reference
$CD8^+$ T cells	Xiao et al.
Naive $CD4^+$ T cells	Xiao et al.
Resting memory $CD4^+$ T cells	Xiao et al.
Activated memory $CD4^+$ T cells	Xiao et al.
Follicular helper T cells	Xiao et al.
Regulatory T cells	Xiao et al.
γδ T cells	Xiao et al.
Naive B cells	Xiao et al.
Memory B cells	Xiao et al.
Plasma cells	Xiao et al.
Resting NK cells	Xiao et al.
Activated NK cells	Xiao et al.
Monocytes	Xiao et al.
M0 macrophages	Xiao et al.
M1 macrophages	Xiao et al.
M2 macrophages	Xiao et al.
Resting dendritic cells	Xiao et al.
Activated dendritic cells	Xiao et al.
Resting mast cells	Xiao et al.
Activated mast cells	Xiao et al.
Eosinophils	Xiao et al.
Neutrophils	Xiao et al.
Fibroblasts	Xiao et al.
Endothelial cells	Xiao et al.

Supplementary Table S7. Biomarkers or gene signatures for immunotherapy response retrieved from the previous studies.

Immunotherapy response biomarkers	Reference
Integrated cytokine score	Wolf et al.
Chemokine12 score	Wolf et al.
Module5 TcellBcell score	Wolf et al.
STAT1 signature	Wolf et al.
Dendritic cell signature	Wolf et al.
B cell signature	Wolf et al.
Tumor mutation burden	Litchfield et al.
APOBEC signature	Litchfield et al.
T cell inflamed GEP signature	Litchfield et al.
Cytolytic score	Litchfield et al.
CD8 T effector signature	Litchfield et al.
CD8A expression	Litchfield et al.
CXCL9 expression	Litchfield et al.
CD274 expression	Litchfield et al.
CD38 expression	Litchfield et al.
CXCL13 expression	Litchfield et al.
