# Supplementary material for: Single-cell morphological and topological atlas reveals the ecosystem diversity of human breast cancer
Source: Nat Commun. 2023 Oct 25;14:6796. doi: 10.1038/s41467-023-42504-y (PMC10600153; doi:10.1038/s41467-023-42504-y)
Supplement: Supplementary file 3 — Description of Additional Supplementary Files [file 41467_2023_42504_MOESM3_ESM.pdf]

Description of Additional Supplementary Files Document

Supplementary Movie 1 : Video illustrating the functions of our sc-MTOP platform.

Supplementary Software 1 : Source codes for single-cell morphological and topological profiling.

Supplementary Data 1 : Example data of the results of single-cell morphological and topological profiling.
